# Sub-Ångström Three-Dimensional Electron Diffraction Reveals Crystal Structures and Phase Transformations in Liquids

**DOI:** 10.1021/jacs.6c02069

**Published:** 2026-04-17

**Authors:** Huiqiu Wang, Joakim Lajer, Edward T. Broadhurst, Tayyaba Malik, Murat N. Yesibolati, Emil C. S. Jensen, Kristian S. Mølhave, Hongyi Xu, Xiaodong Zou

**Affiliations:** † Department of Chemistry, Stockholm University, Stockholm SE-106 91, Sweden; ‡ DTU Nanolab, National Centre for Nano Fabrication and Characterization, Technical University of Denmark, 2800 Kongens Lyngby, Copenhagen 2800, Denmark; § Insight Chips Aps, 5205 DTU Science Park, 2800 Kongens Lyngby, Copenhagen 2800, Denmark; ¶ Research School of Chemistry, Australian National University, Acton, ACT 2601, Australia

## Abstract

Atomic-scale insights into phase transitions and structural dynamics of crystals in liquids are fundamental for understanding chemical, physical, and biological processes. Liquid-phase transmission electron microscopy (LP-TEM) integrates diffraction, imaging, and spectroscopy and has opened new opportunities to study nanoscale materials in liquid environments. Yet, atomic-scale electron crystallographic analysis of crystals in liquids remains elusive. Here, we establish sub-Ångström liquid-phase three-dimensional electron diffraction (LP-3D ED) for capturing phase transformation and determining atomic crystal structures *in situ* by exploiting nanochannel liquid cells. The well-defined and ultrathin liquid layers confined within the nanochannels enable the acquisition of 3D ED data at 0.80 Å resolution from organic molecular crystals in liquids at room temperature. Using LP-3D ED combined with liquid flow control, we observe the β-to-α phase transformation of glycine and *in situ* crystallization of a novel hydrated aluminum-glycine phase in aqueous solution in the nanochannels. We demonstrate *ab initio* crystal structure determination at sub-Ångström resolution by LP-3D ED, and identify a novel hexanuclear aluminum-hydroxide-glycine cluster in the *in situ* formed aluminum-glycine phase. This work demonstrates the capability of LP-3D ED to probe structural evolution and to reveal solvated crystal structures of nano- and microcrystals directly in liquid environments.

## Graphical abstract

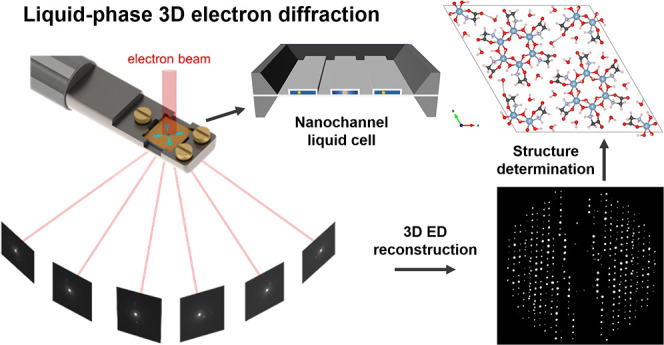

## Introduction

Phase transitions and structural dynamics of crystalline materials in liquid environments are ubiquitous in many chemical processes,^1–7^ including catalysis, guest adsorption, self-assembly, and crystallization. Accurate phase analysis and structure identification under real conditions are therefore essential for revealing native crystal structures and understanding structure–property relationships. Despite their significance, *in situ* phase analysis and *ab initio* crystal structure determination in liquid environments remain challenging.

Liquid-phase transmission electron microscopy (LP-TEM) has emerged as a powerful method for studying nanoscale materials directly in liquid, leveraging diffraction, imaging, and spectroscopy.^8–12^ In a vacuum-sealed liquid cell, the liquid sample is confined between two electron-transparent membranes, thereby allowing the imaging of dynamic processes without freezing or drying. The current LP-TEM imaging platforms have notable limitations. Attaining atomic resolution typically requires highly specialized liquid cells made from two-dimensional materials or polymer membranes,^4,13,14^ which present challenges in versatility and reproducibility. Furthermore, obtaining sufficient imaging resolution necessitates relatively high electron doses (typically thousands of e Å^–2^),^4,15^ leading to radiolysis and beam-induced damage, which is particularly problematic for beam-sensitive materials such as organic and hybrid crystals.

Electron diffraction (ED) offers an attractive alternative. ED requires 2 orders of magnitude lower electron doses (tens of e Å^–2^) than those for TEM imaging, greatly reducing radiation damage while still providing atomic-scale structural information. Besides, achieving atomic structural information through ED is more feasible and less demanding in terms of both instrumentation and technical expertise compared to TEM imaging. Over the past decade, three-dimensional ED (3D ED), analogous to single-crystal X-ray diffraction, has become an emerging technique for resolving 3D atomic structures of nano- and micrometer-sized crystals in dry or cryogenic conditions.^16–23^ However, only very few crystal structural analyses using 3D ED data collected in liquids with clamped chip LP-TEM cells have been reported.^24,25^ This is because obtaining high-resolution 3D ED data has been challenging due to the strong scattering of thick liquid layers around crystals, caused by the bulging of the encapsulating membrane.^9^ Electron beam shower was often needed to partially remove the liquid around the crystals before data collection.^24,25^

Graphene liquid cells (GLCs) pushed liquid-phase imaging to atomic resolution, but challenges remain: reproducibility of encapsulation, lack of flow control, and extreme internal pressures (often >100 MPa) that may alter sample states and radiolytic chemistry.^26–28^ One proof-of-concept study tested GLCs for collecting diffraction data from lysozyme crystals,^29^ but the data resolution was only 3.0 Å, which is not high enough for systematic structure determination.

Recently, nanochannel liquid cells have emerged as a promising platform. Precise wafer-bonded channels with micrometer-scale widths and controlled thickness reduce bulging to <20 nm under TEM vacuum,^30,31^ while supporting liquid flow. These devices combine atomic resolution with stable and reproducible conditions for imaging and trapping nanocrystals.^32^ Yet, their potential for *ab initio* crystal structure determination by liquid-phase electron diffraction has not been assessed.

In this work, we establish sub-Ångström liquid-phase three-dimensional electron diffraction (LP-3D ED) for structure determination of nano- and microcrystals in liquids using nanochannel liquid cells. The capacity of this method is demonstrated by directly observing the humidity-induced β-to-α polymorphic transformation of glycine and by monitoring the reaction between amorphous alumina and glycine in an aqueous solution within the nanochannels, leading to the formation of a previously unknown hydrated aluminum-glycine phase. We show, for the first time, that 3D ED data at sub-Ångström resolution can be collected from organic and hybrid molecular crystals in liquid environments at room temperature, allowing *ab initio* crystal structure determination and accurate localization of all atoms, including hydrogens. Using LP-3D ED, we identify a novel hexanuclear aluminum-hydroxide-glycine ring cluster, (Al­(OH)_2_Gly)_6_, as a main building unit of the hydrated aluminum-glycine structure. These clusters assemble through water-mediated hydrogen bonding into a 3D supramolecular network. These results establish LP-3D ED as a powerful complement to conventional LP-TEM imaging, providing a new route for probing phase transformations and determining atomic-resolution crystal structures in liquid environments.

## Results and Discussion

### Sub-Ångström-Resolution LP-3D ED Data Enabled by Nanochannel Liquid Cell

As shown in Figure 1A, the nanochannel chip in the nanochannel liquid cell integrates four inlets connected to two bypass channels, which are linked by suspended nanochannels that serve as the TEM observation windows. Applying differential pressure across the bypass channels drives the liquid through the nanochannels, enabling controlled flow and maintaining a fresh sample environment. The liquid cell used in this study featured a 2 μm channel width, a ∼172 nm channel height, and 25 nm SiN_*x*_ membranes on both sides (Figures 1B and S1). These nanoscale dimensions minimized electron scattering from liquids, thereby allowing high-resolution diffraction data. Crystals could be grown directly within the channels, after which the chip was mounted in the TEM holder. LP-3D ED data sets were acquired from submicrometer-sized crystals by continuously rotating the sample holder from −30° to 30°, with a cumulative electron dose of approximately 18 e Å^–2^ per data set (Figure 1C), using Instamatic software.^33^ Then, the 3D reciprocal lattice can be reconstructed based on the angular relationship between the ED frames (Figure 1D), allowing for the extraction of unit cell parameters and intensities as well as the determination of reflection conditions and symmetry.

**Figure 1:**
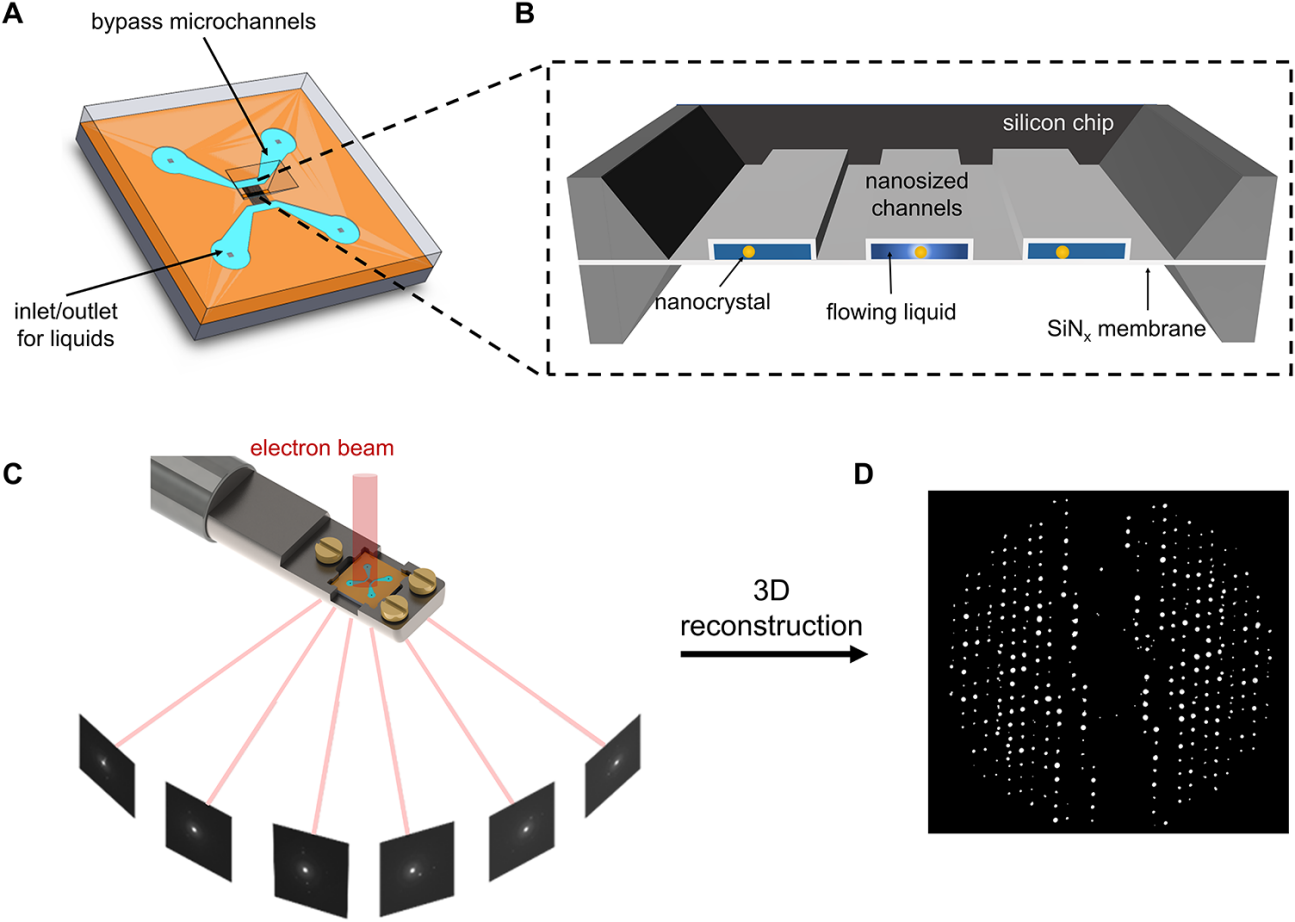
LP-3D ED with sub-Ångström resolution. (A) Representation of the nanochannel chip. (B) Schematic of the nanochannel liquid cell setup. (C) Schematic diagram of collecting the LP-3D ED data set by continuously rotating a fluid flow holder. (D) Reconstructed 3D reciprocal lattice using the LP-3D ED data set.

### Polymorphic Phase Transformation of Glycine

We first applied LP-3D ED to study the polymorphic phase transformation of glycine. Polymorph evolution of glycine during crystal growth was previously studied ex situ by time-resolved 3D ED, where the phase transformation of β-glycine to α-glycine occurred within 1 min.^34^ Dry β-glycine crystals were prepared by evaporating a saturated glycine solution in the nanochannels. The resulting plate-like crystals are attached to one side of the channel wall and grown along the channel direction (Figures 2A and S2). 3D ED data were collected from three crystals before introducing glycine solution (Figure S3), from which the unit cell parameters were determined to be *a* = 5.21(3) Å, *b* = 6.31(2) Å, *c* = 5.59(4) Å, α = 90°, β = 112.3(6)°, and γ = 90° (Table S1), which agree with those of β-glycine with the space group *P*2_1_.

**Figure 2:**
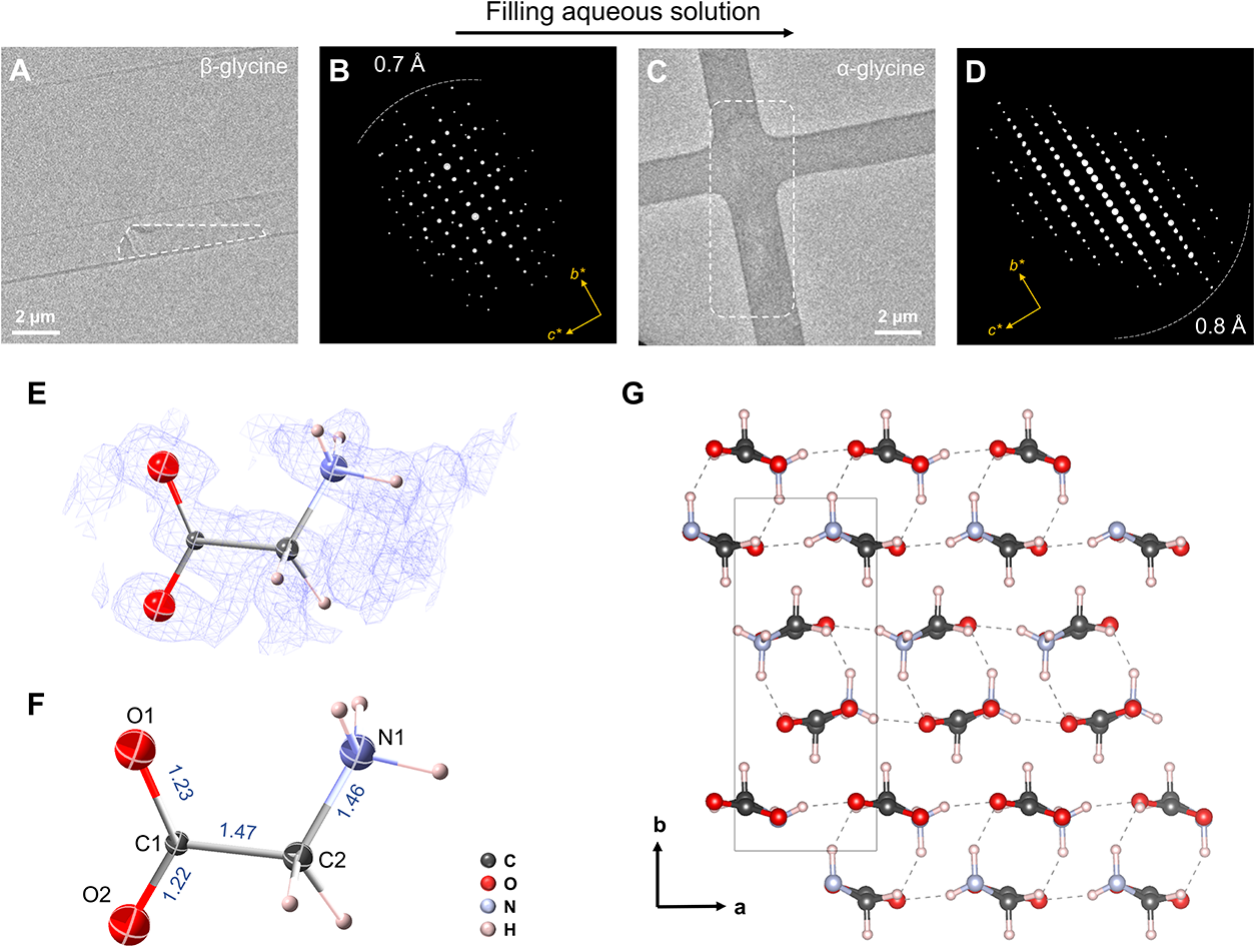
Revealing phase transformation of glycine crystal polymorphs in liquids. (A) TEM image of a dry β-glycine crystal. (B) Reconstructed 3D reciprocal lattice of the crystal in (A). (C) TEM image of a wet α-glycine crystal in an aqueous solution. The image contrast of the crystals in solution is significantly reduced, and the shape of the crystals is more difficult to discern compared to that in (A). (D) Reconstructed 3D reciprocal lattice of the crystal in (C). (E) Observed Fourier map for the asymmetric unit of α-glycine (isosurface level: 0.40 σ). (F) Asymmetric unit of the α-glycine crystal structure was determined using LP-3D ED data. The bond lengths are given in ångströms (Å). (G) The crystal structure of α-glycine was determined by LP-3D ED.

When saturated glycine solution was reintroduced through the bypass channels in the chips, crystals with different morphologies formed rapidly (within 1–2 min) in the nanochannels, fully immersed in liquid (Figures 2C and S4). LP-3D ED data were collected from four wet crystals, from which the unit cell parameters were determined to be *a* = 5.035(9) Å, *b* = 11.80(5) Å, *c* = 5.30(1) Å, α = 90°, β = 111.7(3)°, and γ = 90° (Table S1). Based on diffraction intensities and reflection conditions (Figures S3), the crystals are monoclinic with the space group *P*2_1_/*n*, which agrees with those of α-glycine. This indicates that a phase transformation from β-glycine to α-glycine occurred, consistent with literature reports.^35–37^ The distinct morphologies of α- and β-glycine observed within the nanochannels support a solution-mediated phase transformation mechanism rather than a direct solid–solid transition.

It is worth noting that the α- and β-glycine crystals grown within the nanochannels exhibit morphologies distinct from those crystallized directly on the EM grid. In the latter case, α-glycine forms large (>5 μm) plate-like crystals, whereas β-glycine adopts a characteristic “shark’s tooth” morphology.^34^ In contrast, confinement in the nanochannels leads to clearly different crystal habits for both polymorphs.

Due to the restricted tilting angle (±30°) and preferred orientation of plate-like crystals, completeness from individual data sets was low. To address this, four consistent α-glycine data sets were merged, yielding 74.7% completeness to 0.80 Å resolution. To the best of our knowledge, this represents the highest resolution reported for crystals in liquids to date (Table S1).^24,25,29^ Such high-resolution diffraction data provide a robust foundation for atomic-scale 3D structure determination. The structure of α-glycine was subsequently solved *ab initio* from the merged LP-3D ED data using direct methods in SHELXT^38^ in the space group *P*2_1_/*n* (Figure 2G). All nonhydrogen atoms (C, N, and O) are clearly resolved from the Fourier maps (Figure 2E). During structure refinement,^39^ five symmetry-independent hydrogen atoms were also located by combining Q peaks and chemical knowledge. Each nitrogen atom bonds to three hydrogen atoms, indicating that the nitrogen atom was protonated. The refined chemical composition is C_2_H_5_NO_2_, and the refinement converged to *R*_1_= 0.2331 for 333 reflections with *F*_o_ > 4σ­(*F*_o_). The obtained bond lengths of O1–C1, O2–C1, N1–C2, and C1–C2 are 1.23(1), 1.22(1), 1.46(1), and 1.47(1) Å, respectively (Figure 2F), which are very similar (within 0.07 Å root-mean-square deviation) to the reported values determined by single-crystal X-ray diffraction data (SCXRD)^40^ (Table S3). Thus, LP-3D ED is a reliable method for determining the 3D atomic structure of crystals in liquids. For dry β-glycine, the merged data completeness (∼37%) was insufficient for *ab initio* solution, but refinement using the SCXRD structure as a starting model confirmed the observed phase (Figure S5).

Notably, the crystallographic figures of merit (e.g., *R*_int_ and *R*_1_) for LP-3D ED are comparable to those of cryogenic 3D ED (Table S6).^34^ The required dose (18 e Å^–2^) lies well within the “low-dose” regime (<100 e Å^–2^),^41^ minimizing radiation damage. Furthermore, the liquid flowing within the nanochannels prevents the accumulation of radiolytic byproducts in nanochannels, alleviating the effect of radiation damage and bubble formation. Under these experimental conditions, no bubble formation was observed (Figure S6). The ability to achieve sub-Ångström resolution under such conditions demonstrates that electron-beam-induced alterations are negligible, enabling reliable atomic-scale structural analysis directly in liquids.

### *In Situ* Crystallization and Structure of a Novel Hydrated Aluminum-Glycine Phase

We further explored the use of nanochannels for monitoring crystal growth and subsequent *ab initio* structure determination of previously unknown phases directly in liquid environments. For this, we employed a nanochannel liquid cell coated with a thin amorphous alumina layer introduced by atomic layer deposition (ALD) during the manufacture. Energy-dispersive spectroscopy (EDS) mapping confirmed the presence of Al within the nanochannels (Figure S7). *In situ* crystallization was achieved by heating a saturated glycine solution at 80 °C inside of the alumina-coated nanochannels, followed by refilling. We revealed block-like crystals grew from the channel wall, submerged in solution (Figure S8).

LP-3D ED data were collected from seven crystals, from which the unit cell parameters were determined to be *a* = 24.30(5) Å, *b* = 24.30(5) Å, *c* = 4.94(1) Å, α = 90°, β = 90°, and γ = 120° (Table S4). The space group was determined from the diffraction intensities and reflection conditions to be *R*-3 (no. 148) (Figure S9). Electron energy loss spectroscopy (EELS) showed the presence of Al in the crystals (Figure S10), indicating that the crystals may be aluminum-glycine compound.

Two high-quality LP-3D ED data sets from two different crystals (Figure S8) were merged, achieving a completeness of 94.9% due to the high symmetry (Table S5). The crystal structure of the aluminum-glycine compound was solved ab initio and subsequently refined (Figure 3). All nonhydrogen atoms (C, N, O, and Al), including one water oxygen, were successfully resolved in the Fourier map (Figure 3A). All eight symmetry-independent hydrogen atoms could be identified from difference Fourier maps and refined. The refinement converged to *R*_1_ = 0.1542 for 317 reflections with *F*_o_ > 4σ­(*F*_o_) and *R*_1_ = 0.2412 for all 657 reflections (Table S5).

**Figure 3:**
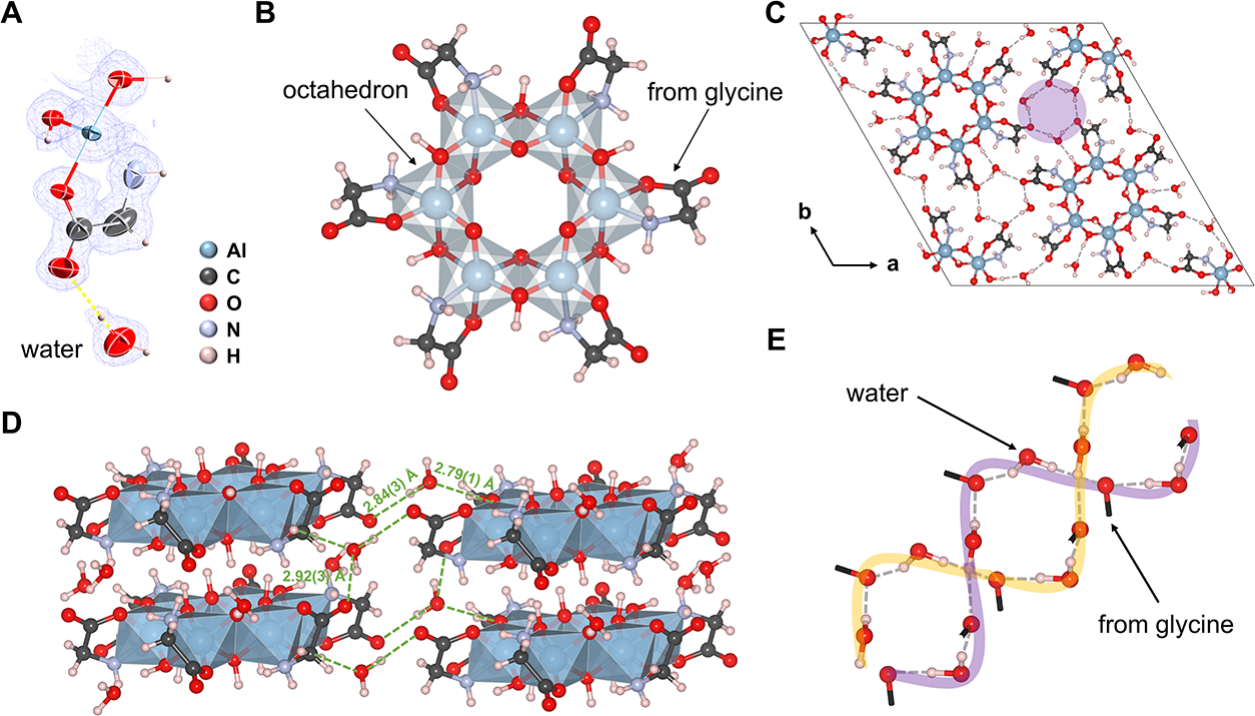
Novel hydrated aluminum-hydroxide-glycine crystal structure discovered by *in situ* LP-3D ED. (A) The asymmetric unit of the hydrated aluminum-hydroxide-glycine cluster is superimposed on the Fourier map (isosurface level: 0.21 σ). (B) The hexanuclear aluminum-hydroxide-glycine cluster built from six edge-sharing Al­(OH)_4_Gly octahedra forming a six-membered ring cluster, (Al­(OH)_2_Gly)_6_. Each aluminum atom is chelated by a glycine molecule via its O and N atoms. (C) The refined three-dimensional atomic structure of the hydrated aluminum-glycine crystal viewed along the *c*-axis. A purple circle marks the projection of a double-helical hydrogen-bonding chain along the *c*-axis. (D) Packing of the (Al­(OH)_2_Gly)_6_ rings, where water molecules bridge the neighboring rings through hydrogen bonding (O···HO), indicated as green dashed lines together with the O···O distances. (E) The double-helical hydrogen-bonding chains. Two color stripes are added for eye guidance of the chains.

The aluminum-glycine structure consists of hexanuclear aluminum-hydroxide-glycine clusters (Al­(OH)_2_Gly)_6_ that are interconnected by water molecules. Each cluster features a six-membered ring formed by six edge-sharing Al­(OH)_4_Gly octahedra, where each aluminum atom is chelated by a glycine molecule via its O and N atoms (Figure 3B). These (Al­(OH)_2_Gly)_6_-rings are held together via water molecules through OH···O hydrogen bonding to form a three-dimensional hydrogen-bonded supramolecular network (Figure 3C,D). The (Al­(OH)_2_Gly)_6_-rings are stacked along the *c*-axis to form one-dimensional (1D) tubes (Figure 3C). Water molecules occupy the gaps between the tubes, each forming three hydrogen bonds with two (Al­(OH)_2_Gly)_6_-rings. This arrangement synergistically links the 1D tubes into a stable network structure. Remarkably, within the confined intertube space, the water molecules and carbonyl groups form double-helical hydrogen-bonding chains of opposite handedness along the *c*-axis (Figure 3D,E).

Using LP-3D ED, we discovered an unprecedented hexanuclear aluminum-hydroxide-glycine ring (Al­(OH)_2_Gly)_6_ cluster in situ formed in the nanochannel liquid cell. Aluminum clusters are often found as building units in metal–organic frameworks. We envisage that our discovery may inspire the design of new metal–organic frameworks and supramolecular assemblies.

Overall, the LP-3D ED represents a challenging but transformative advance in in situ electron microscopy. Looking forward, LP-3D ED could be applied to a wide range of systems to probe responses to external stimuli, including temperature, electric or magnetic fields, host–guest interactions, and ligand coordination, and can be integrated with complementary approaches such as serial electron diffraction (SerialED).^42,43^

It is worth mentioning that the current nanochannel liquid cell design restricts a maximum tilt from −30° to 30° in a standard TEM, limiting data completeness for low-symmetry crystals such as α- and β-glycine. While merging data sets from multiple crystals offers partial solutions, this approach depends on crystal orientation within the nanochannels. For in situ grown crystals with strong orientation preferences, a limited tilt range can hinder the ab initio structure determination. Improvements on designs that permit >65° rotation are being tested to expand the possibilities with LP-3D ED. In addition, incorporating trapping regions into the channels, as recently demonstrated,^32^ could allow random orientation of nanocrystals delivered at low concentration without clogging, further improving data quality.

## Conclusions

In summary, we demonstrate that LP-3D ED enables sub-Ångström structure determination of nano- and microcrystals directly in liquid environments. This capability allowed us to capture, for the first time, the β-to-α transformation of glycine and to follow the growth of a novel hydrated aluminum-glycine phase. The atomic structure of this new phase was solved *ab initio* from LP-3D ED data collected from crystals immersed in aqueous solution. All atoms, including hydrogens, could be located and refined. The discovery of the unprecedented hexanuclear aluminum-hydroxide-glycine cluster further demonstrates the strength of LP-3D ED in revealing solvent-stabilized structures in their native state that are inaccessible or altered under cryogenic conditions. By extending electron crystallography to realistic liquid environments, LP-3D ED provides a powerful platform for probing structural evolution and for discovering solvated nano- and microcrystals across chemistry, biology, and materials science.

## Supplementary Material



## Notes

Experimental details, structural analysis by 3D ED, and other characterizations (PDF)
