# Supplementary material for: Sub-Ångström Three-Dimensional Electron Diffraction Reveals Crystal Structures and Phase Transformations in Liquids
Source: J Am Chem Soc. 2026 Apr 17;148(16):17174–80. doi: 10.1021/jacs.6c02069 (PMC13133786; doi:10.1021/jacs.6c02069)
Supplement: Supplementary file 1 [file ja6c02069_si_001.pdf]

Supplementary Information for

Sub-Ångström three-dimensional electron diffraction reveals crystal structures and phase transformations in liquids

Authors: Huiqiu Wang,[1]†, Joakim Lajer[2]†, Edward T. Broadhurst[1], Tayyaba Malik[2], Murat N. Yesibolati[2], Emil C. S. Jensen[3], Kristian S. Mølhave[2]*, Hongyi Xu[1,4]*, Xiaodong Zou[1]*

Affiliations:

[1]Department of Chemistry, Stockholm University, Stockholm, SE-106 91, Sweden.

[2]DTU Nanolab, National Centre for Nano Fabrication and Characterization, Technical University of Denmark, 2800 Kongens Lyngby, Copenhagen, Denmark.

[3]Insight Chips Aps, DTU Science Park, 2800 Kongens Lyngby, Copenhagen, Denmark.

[4]Research School of Chemistry, Australian National University, Acton, ACT 2601, Australia

†These authors contributed equally to this work.

*Corresponding email: krmo@dtu.dk; Hongyi.Xu1@anu.edu.au; xiaodong.zou@su.se

This PDF file includes:

Supplemental experimental procedures

Figures S1 to S10

Tables S1 to S7

Supplemental References

Supplemental methods

Preparation of α-glycine and β-glycine microcrystals

Commercial glycine (Sigma-Aldrich ACS reagent ≥ 98.5%) was dissolved in Milli-Q water to prepare a 50% saturated glycine solution for liquid cells. 0.2 µL of a 50% saturated glycine solution was injected into the inlets of one bypass channel and then blotted, leaving the channels filled with the solution. The chip was then placed on a hotplate for 2 minutes at 80°C to trigger the crystallization process. Subsequently, the inlets to the other bypass were punctured and the chip was placed in the holder, using the empty bypass port to refill the saturated glycine solution. Initial crystallization from the hotplate was checked optically before loading the chip into the holder.

Preparation of the aluminum-glycine crystal

The same procedure as above was implemented, with the only change being the chip used. The channel height is 180 nm, and a 25 nm SiN_x membrane, and a few atomic layers of AlO_x are used for binding.[1] The presence of glycine crystals was checked using optical microscopy before loading into the liquid cell holder.

3D ED Data Collection

Electron diffraction experiments were performed on a Thermo Fisher Themis TEM operating at 300 kV (λ = 0.0197 Å) equipped with a CMOS detector (OneView, 1024 × 1024 pixels, Gatan). 3D ED data were acquired using a selected area aperture on individual crystals in channels, with crystal lengths ranging from 1 to 4 µm. A TEM holder enabling liquid flow obtained from *Insight Chips* was used for the analysis, shown in Figure S1. The TEM holder has four tubes to control flow in two bypass microchannels connecting to the nanochannels on the suspended membrane imaging region with crystals inside the channels.

All 3D ED data were collected at room temperature while rotating the goniometer continuously between -30 and 30° in the α tilt axis, limited by the pole-piece geometry of the TEM. The exposure time (0.96 s) and rotation speed (0.1 °/s) were chosen so that individual diffraction frames were integrated over 0.096° of reciprocal space. The estimated dose rate was 0.089 e $Å^{-2}$ s^{-1}, with each collection taking on average 200 s, the total estimated dose per 3D ED data collection was 17.8 e $Å^{-2}$.

Data Processing and Structure Determination

REDp was used to determine the unit cell and space group from 3D ED data.[2] Data reduction, integration, and merging were performed with XDS.[3] Structures were solved via direct methods using SHELXT[4] and the kinematic structure refinement was conducted using SHELXL in the

Shelxle interface.[5] Atomic scattering factors for electrons were used. All non-H atoms in the hydrated aluminum-glycine structure were refined anisotropically without any restraints, while those in the α-glycine structure were refined isotropically to avoid negative atomic displacement parameters (ADPs). Due to the low completeness of the β-glycine 3D ED data, it was not possible to perform *ab initio* structure solution. Instead, a standard β-phase structure was used as a starting model for the refinement against the 3D ED data.

Other characterizations

To show the presence of an aluminum oxide layer in the nanochannel, energy-dispersive spectroscopy (EDS) was carried out on an FEI Tecnai T20 at 200 kV. First, the TEM was set up to scanning transmission electron microscopy (STEM) mode to control the scanning beam for the analysis. Point measurements at different α tilt angles were made without recording spectra to get sufficient counts for detection. An α of -25° was found to give the most counts. The reason for this was that at higher α, the Si wedge within the nanochannel chip would tend to block X-rays being produced, causing a reduction in the amount of X-rays hitting the EDS detector at higher α tilt angles. The spot size was set to 6, to provide a high enough count of X-rays to give a signal with a sufficiently high resolution. EDS spectra were taken over the whole nanochannels, and the presence of aluminum, oxygen, silicon and nitrogen was confirmed.

For the chemical analysis of aluminum-glycine crystals, electron energy-loss spectroscopy (EELS) was applied. The EELS data were collected in STEM mode using the Gatan Quantum GIF with a 29.5 mm camera length, a 5 mm entrance aperture, and a collection semi-angle of 28.24 mrad. The low-loss and high-loss spectra were captured simultaneously in dual-EELS mode. The SNR mode was used for STEM-EELS to get high signal-to-noise ratio data. The exposure time for each scanning pixel is 0.05 s with spatial drift correction every 5 rows.

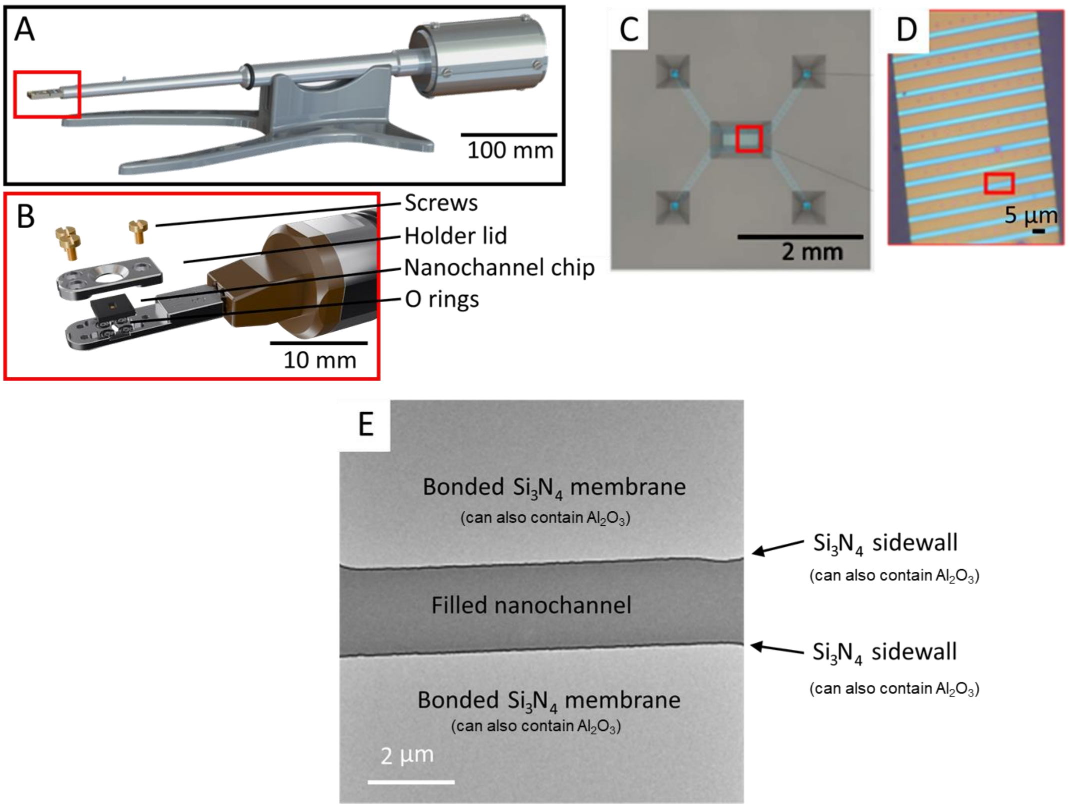

Figure S1. Schematic of the liquid cell chip and holder used in this work. (A) Thermo Fisher Scientific-type liquid cell TEM holder. (B) Expanded view of the tip showing how the liquid chip is held for TEM use. (C) Top-down view of the liquid chip showing the two inlet and outlet ports, bypass microchannels indicated by blue, and a viewing window with suspended nanochannels in the centre. (D) Optical microscopy image of the viewing window, the nanochannels are shown in blue and the partitions in yellow. (E) TEM image of the nanochannel liquid cell. The darker contrast in the middle indicates the nanochannel is filled by liquid.

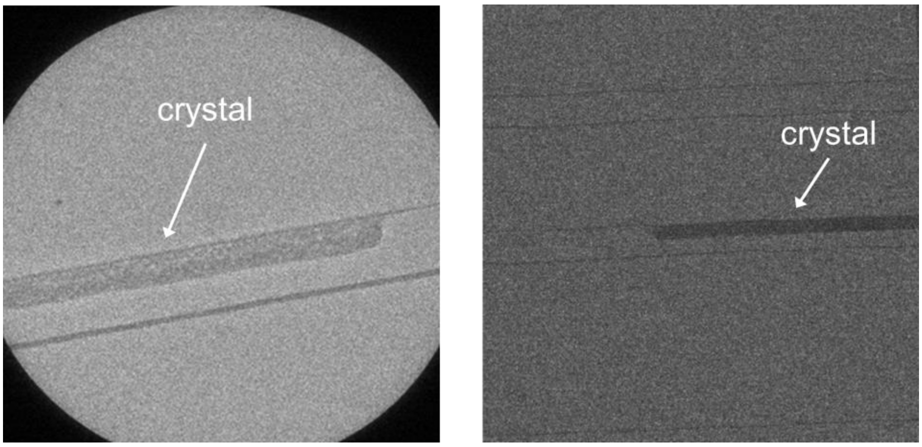

Figure S2. TEM image of a β-glycine crystal in a dry nanochannel.

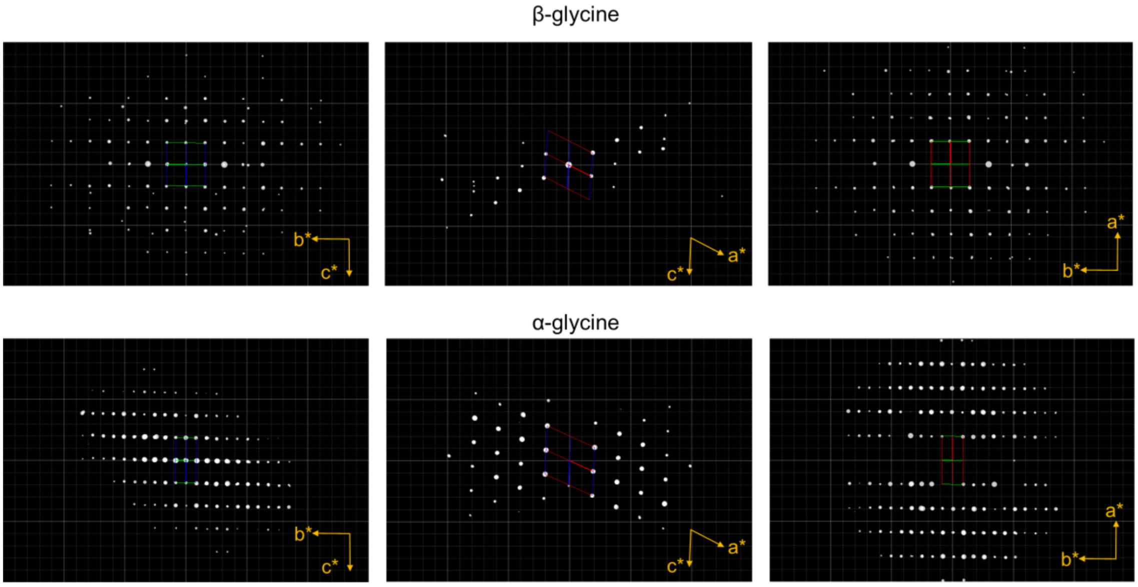

Figure S3. 3D reciprocal lattice of β-glycine (above) and α-glycine (below) reconstructed from 3D ED and LP-3D ED data, respectively.

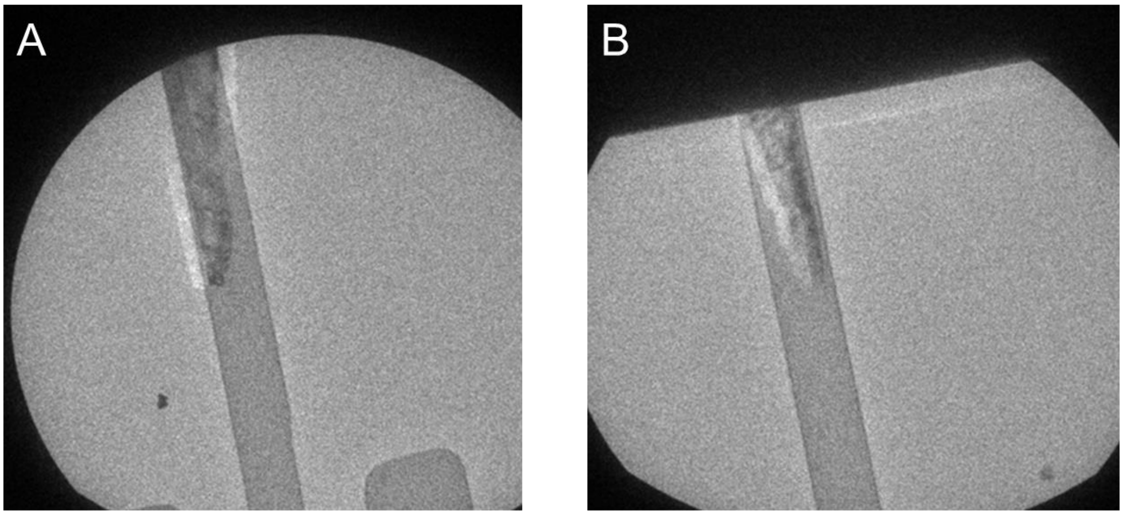

Figure S4. TEM images of glycine crystals in wet nanochannels.

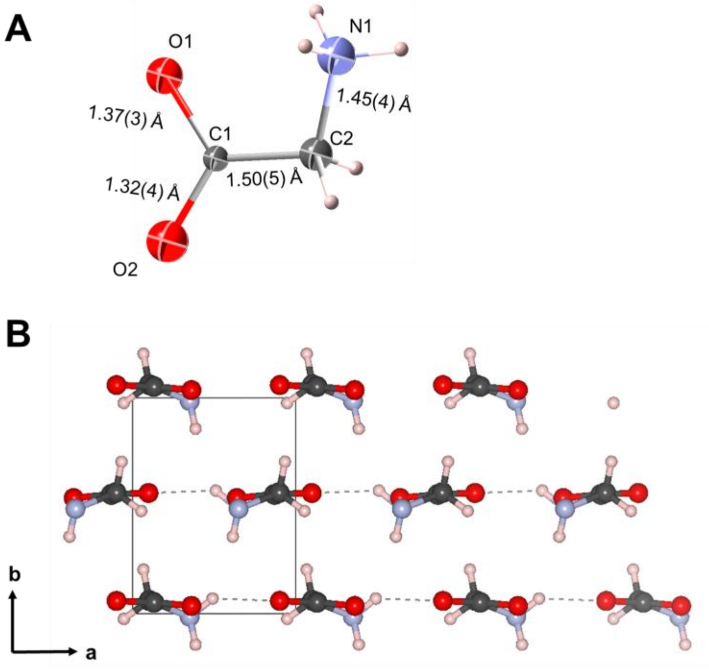

Figure S5. The structure of β-glycine refined against 3D ED data. (A) The asymmetric unit with the bond lengths indicated. (B) Hydrogen-bonding network in β-glycine. Note: Isotropic refinement was conducted.

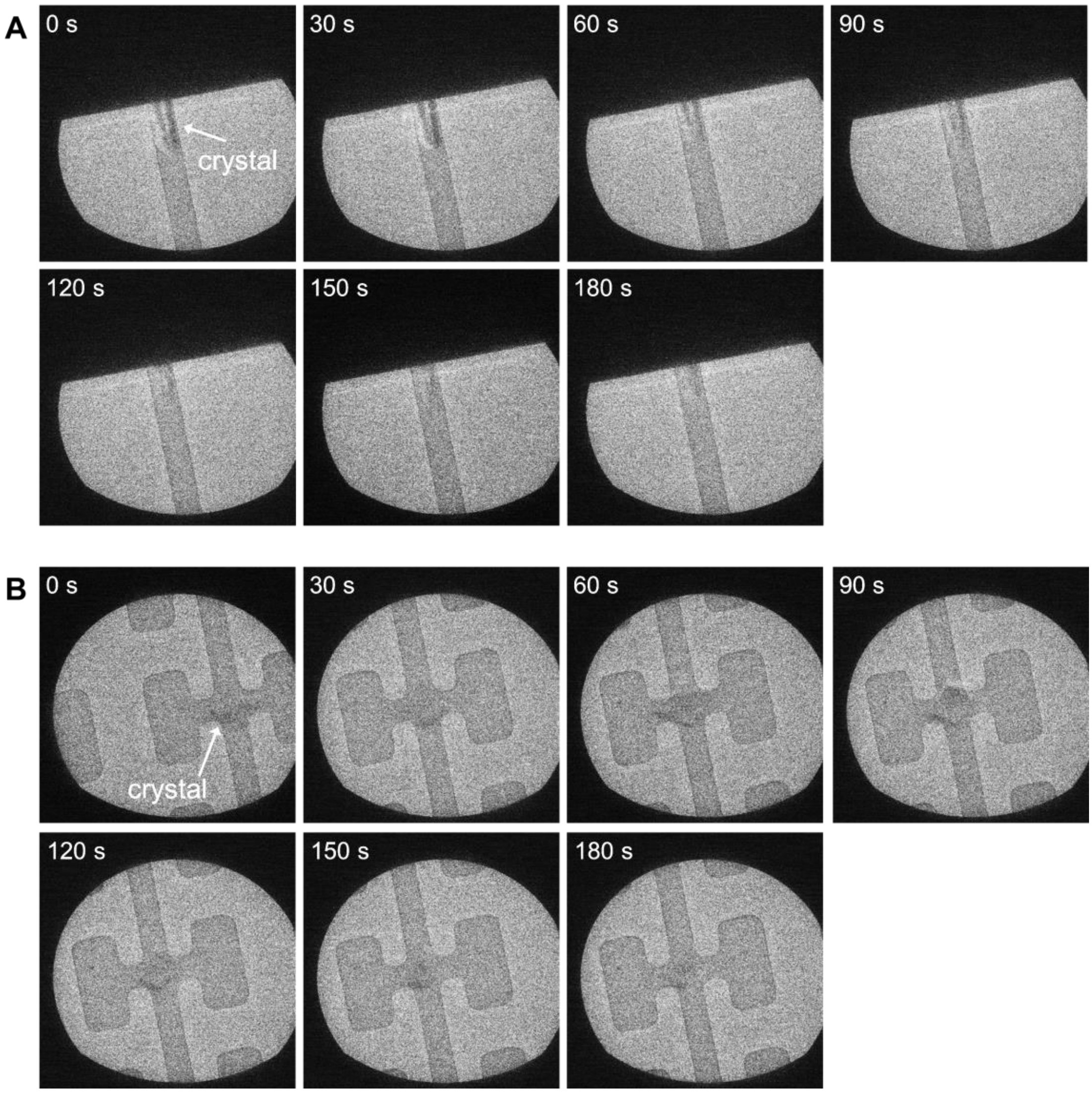

Figure S6. Effects of electron irradiation on α-glycine crystals in liquid confined within nanochannels. The electron dose and illumination conditions were identical to those used for LP-3D ED data collection. Images were acquired at 30 s intervals. No bubble formation was observed over 180 s of continuous irradiation, a duration comparable to that used for a typical LP-3D ED experiment

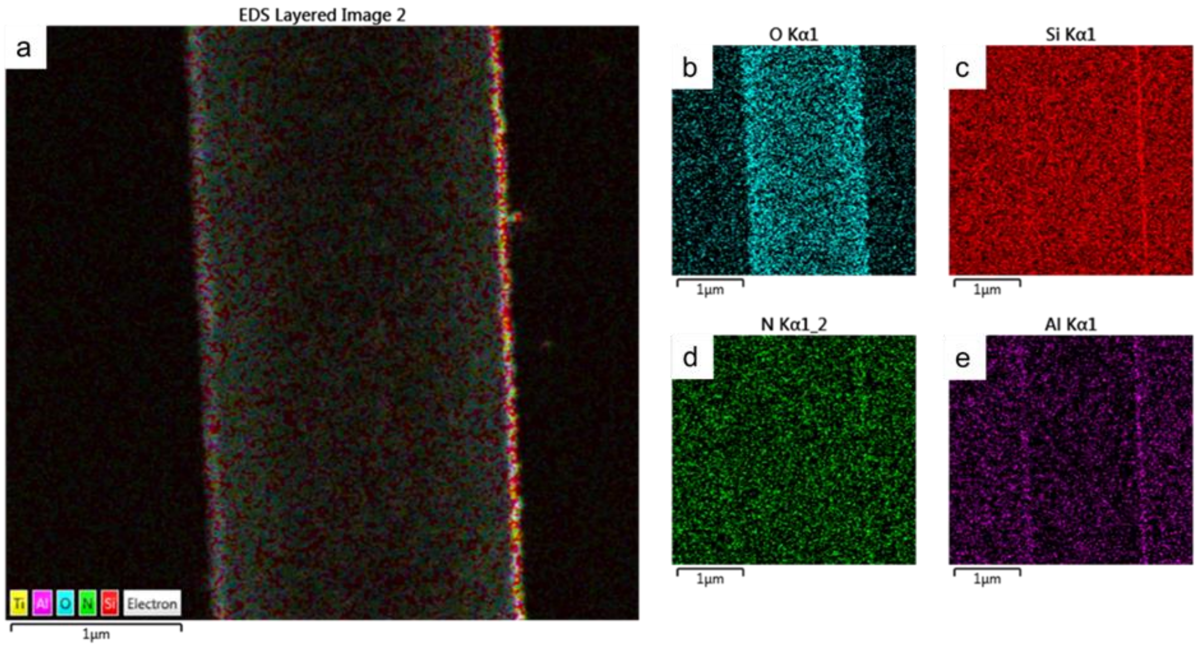

Figure S7. EDS mapping of a nanochannel on the chip. (A) STEM image of the channel with the different atomic spectra overlaid. (b) O Kα1. (c) Si Kα1. (d) N Kα1_2. (e) Al Kα1.

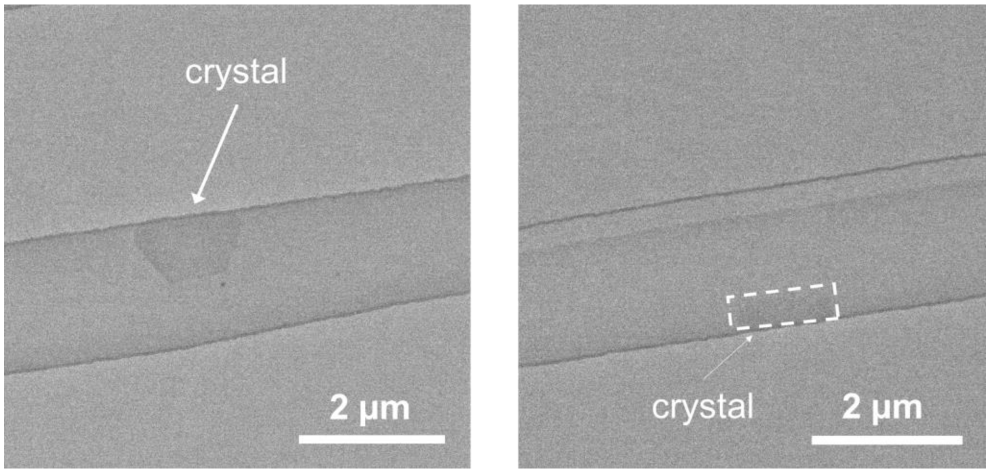

Figure S8. TEM images of hydrated aluminum-glycine crystals in aqueous solution.

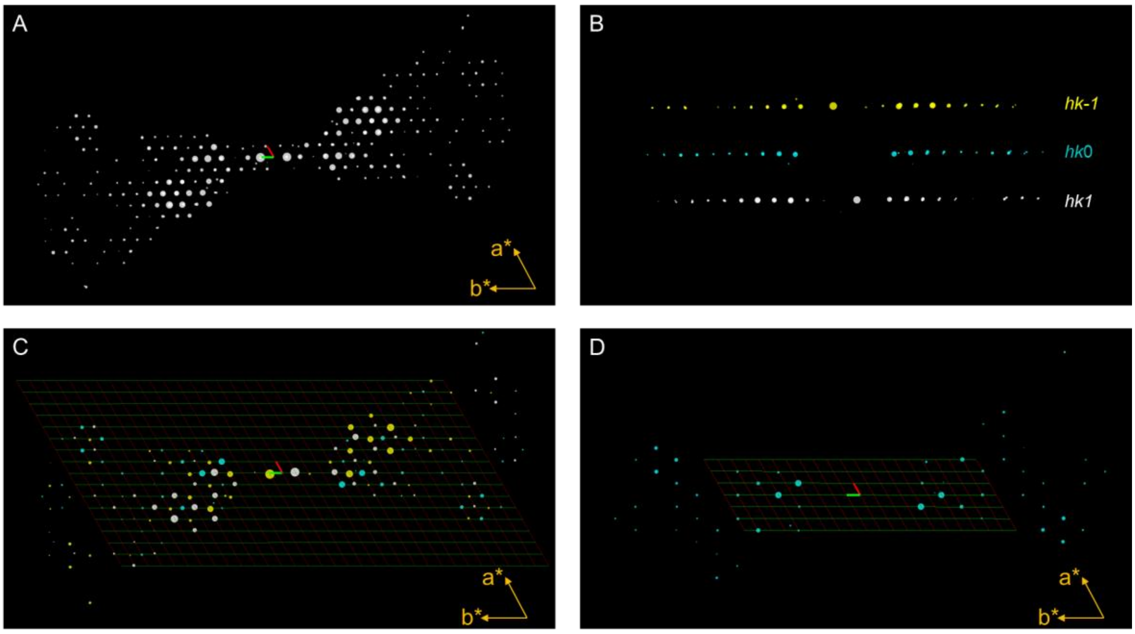

Figure S9. 3D reciprocal lattice of a hydrated aluminum glycine crystal reconstructed from the LP-3D ED data. (A) 3D reciprocal lattice viewed along the *c*-axis. (B-C) Three 2D slices at the (*hk*0), (*hk*-1), and (*hk*1) planes viewed along the (B) *a*-axis and (C) *c*-axis. (D) The 2D slice at the hk0 plane viewed along the *c*-axis. The diffraction spots in the (*hk*-1), (*hk*0) and (*hk*1) planes are shifted by *a** relative to each other, indicating a rhombohedral (*R*) lattice with the reflection condition *hkl*: $-h + k + l = 3n$.

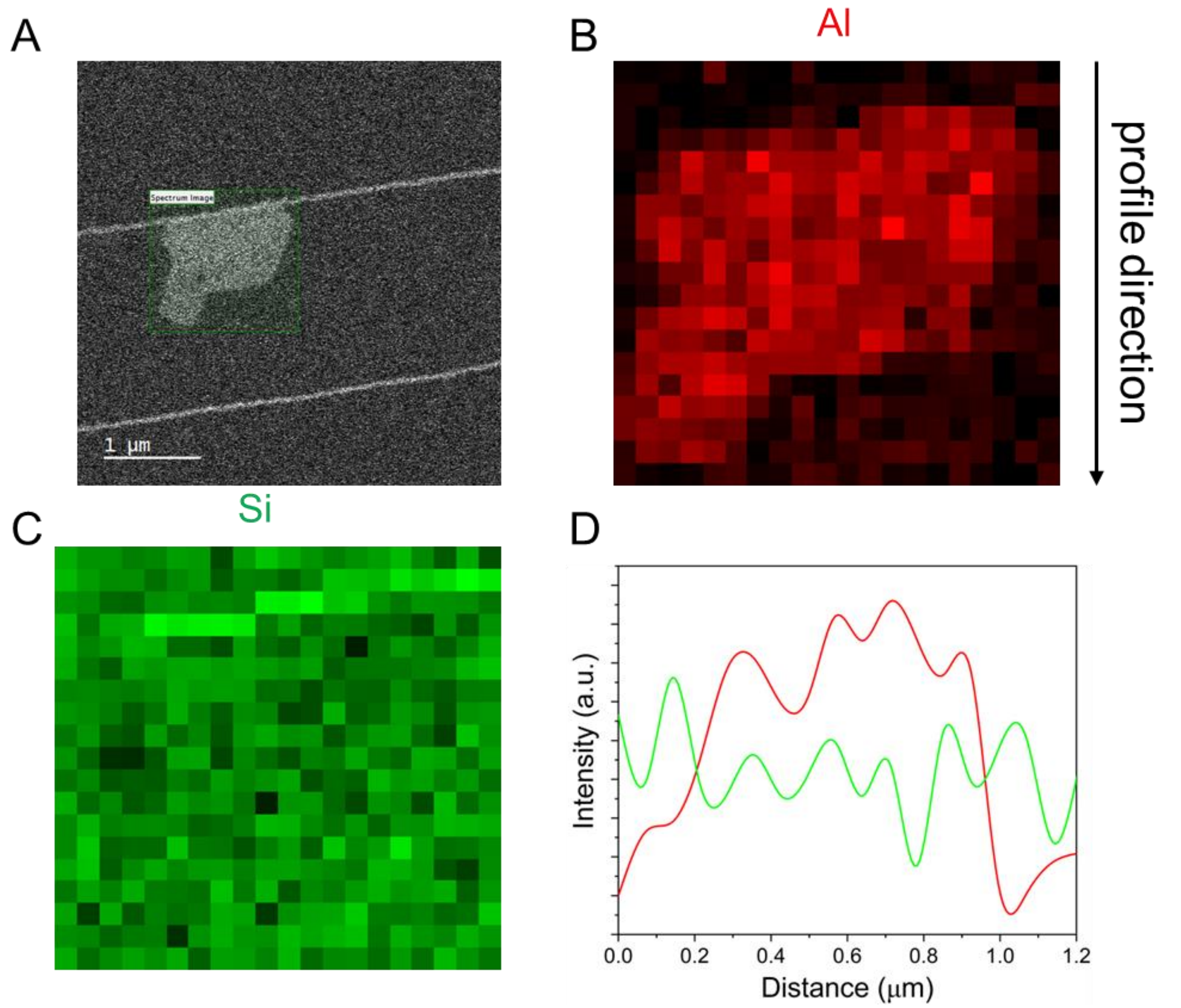

Figure S10. EELS-STEM analysis of an aluminum-glycine crystal. (A) STEM image of the crystal in a nanochannel. (B-C) EELS mapping of Al and Si of the region marked in (A). (D) Profile analysis of the mapping region. The Al signal is concentrated in the crystal while Si is more evenly distributed.

Table S1. The unit cell parameters of β-glycine ($P2_1$) and α-glycine ($P2_1/n$) crystals in dry and aqueous environments, respectively, determined by 3D ED.

Phase	a/Å	b/Å	c/Å	α/°	β/°	γ/°
β	5.18(3)	6.276(8)	5.41(6)	90	111.9(7)	90
	5.26(1)	6.39(1)	5.45(1)	90	112.7(1)	90
	5.26(1)	6.36(1)	5.593(5)	90	112.7(2)	90
α	5.048(6)	11.74(3)	5.41(1)	90	110.9(6)	90
	5.05(1)	11.72(4)	5.41(2)	90	111.1(6)	90
	5.01(1)	11.97(3)	5.285(1)	90	111.2(1)	90
	5.03(2)	11.87(4)	5.353(5)	90	112.6(1)	90

Table S2. LP-3D ED data information and structure refinement (isotropic) of α-glycine.

Data	
Temperature (K)	293
Radiation (Å)	Electrons, 0.01969
Number of crystals	4
Resolution (Å)	0.80
Completeness (%)	74.7
Total, unique reflection,	2019, 459
Rint	0.2635
Refinement	
$N_{reflections}$, $N_{parameters}$, $N_{restraints}$	459, 41, 0
R_1, wR_2 [Fo > 4σ(Fo)]	0.2331, 0.4739
R_1 (all data)	0.2443, 0.4444

Table S3. Comparison of the bond lengths of the α-glycine structure determined by LP-3D ED (this work) and that in the Cambridge Structure Database (CSD, GLYCIN29).[6] The root-mean-square deviation (RMSD) is 0.07 Å.

Bond	LP-3D ED (Å)	GLYCIN29 (Å)
O1-C1	1.23(1)	1.2550(11)
O2-C1	1.22(1)	1.2518(10)
N1-C2	1.46(1)	1.4778(11)
C1-C2	1.47(1)	1.5269(10)

Table S4. The unit cell parameters of seven aluminum-glycine crystals determined from the LP-3D ED data. The space group was determined to be *R*-3.

Dataset	a/Å	b/Å	c/Å	α/°	β/°	γ/°
1	24.6(1)	24.6(1)	4.915(9)	90	90	120
2	24.21(1)	24.21(1)	4.933(5)	90	90	120
3	24.18(1)	24.18(1)	4.872(2)	90	90	120
4	24.49(8)	24.49(8)	4.97(1)	90	90	120
5	24.24(5)	24.24(5)	4.946(3)	90	90	120
6	24.39(7)	24.39(7)	4.941(4)	90	90	120
7	24.2(2)	24.2(2)	4.934(2)	90	90	120

Table S5. Data information and structure refinement (anisotropic) results of LP 3D ED aluminum-glycine crystals.

Data	
Temperature (K)	293
Radiation, wavelength (Å)	Electrons, 0.01969
Number of crystals	4
Resolution (Å)	0.90
Completeness (%)	94.9
Total, unique reflection	1887, 657
R_{int}	0.2311
Refinement	
$N_{reflections}$, $N_{parameters}$, $N_{restraints}$	656, 98, 2
R_1, wR_2 [$Fo > 4\sigma(Fo)$]	0.1542, 0.4983
R_1 (all data)	0.2412

Table S6. Comparison of the crystallographic figure of merits (R-values) of α-glycine crystals.

	LP-3D ED	Cryo-ED[7]
R_{int}	0.2635	0.318
R_1	0.2331	0.219

Supplemental References

1. Laganá, S., Mikkelsen, E.K., Marie, R., Hansen, O., and Mølhave, K. Direct bonding of ALD Al2O3 to silicon nitride thin films. Microelectron. Eng. 176, 71-74 (2017).
2. Wan, W., Sun, J., Su, J., Hovmöller, S., and Zou, X. Three-dimensional rotation electron diffraction: software RED for automated data collection and data processing. J. Appl. Crystallogr. 46, 1863-1873 (2013).
3. Kabsch, W. XDS. Acta Crystallogr., Sect. D: Biol. Crystallogr. 66, 125-132 (2010).
4. Sheldrick, G.M. SHELXT - integrated space-group and crystal-structure determination. Acta Crystallogr., Sect. A: Found. Adv. 71, 3-8 (2015).

5. Sheldrick, G.M. Crystal structure refinement with SHELXL. Acta Crystallogr., Sect. C: Struct. Chem. 71, 3-8 (2015).
6. Iitaka, Y. (1960). The crystal structure of β-glycine. Acta Crystallogr. 13, 35-45.
7. Broadhurst, E.T., Xu, H., Clabbers, M.T.B., Lightowler, M., Nudelman, F., Zou, X., and Parsons, S. (2020). Polymorph evolution during crystal growth studied by 3D electron diffraction. IUCrJ 7, 5-9.
